# Photodynamic impact application in children with spine deformities: encouraging results

**DOI:** 10.1186/1748-7161-10-S1-P8

**Published:** 2015-01-19

**Authors:** Shashko Aleksei, Kurchenko Sergey

**Affiliations:** 1 Children's Rehabilitation Center of Orthopedics and Traumatology "Ogonyok", St. Petersburg, Russia

## Introduction

As was shown, the targets for therapeutic intervention in treatment of AIS are growth plates (GP) of the vertebral bodies, which are intensively proliferating cells. An encouraging method to control GP activity is a photodynamic impact (PDI). It is based on the arising of singlet oxygen and active radicals under the influence of laser irradiation on proliferating cells, that have accumulated photosensitizer (PS), and ultimately leads to their necrosis and apoptosis. In our previous histological studies it was found that PS actively accumulates in GP cells of long tubular bones and PDI on them causes their decreasing and reducing of the chondrocytes total number in them. This suggests a similar effect of PDI on vertebral bodies GP, since they are histologically identical to former ones.

## Objectives

1 Confirm clinical effect of PDI on GP at the macroscopic level.

2 Adapt PDI for treatment of the spine deformities in children.

## Material and methods

**Figure 1:**
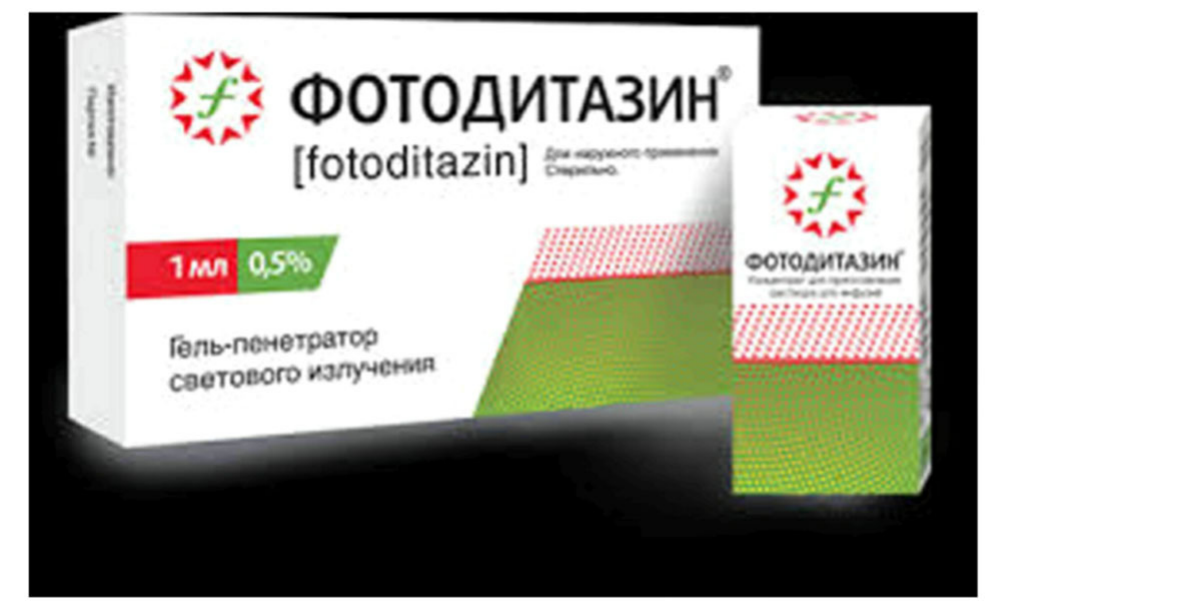
Fotoditazin ®, gel penetrator of light radiation

**Figure 2:**
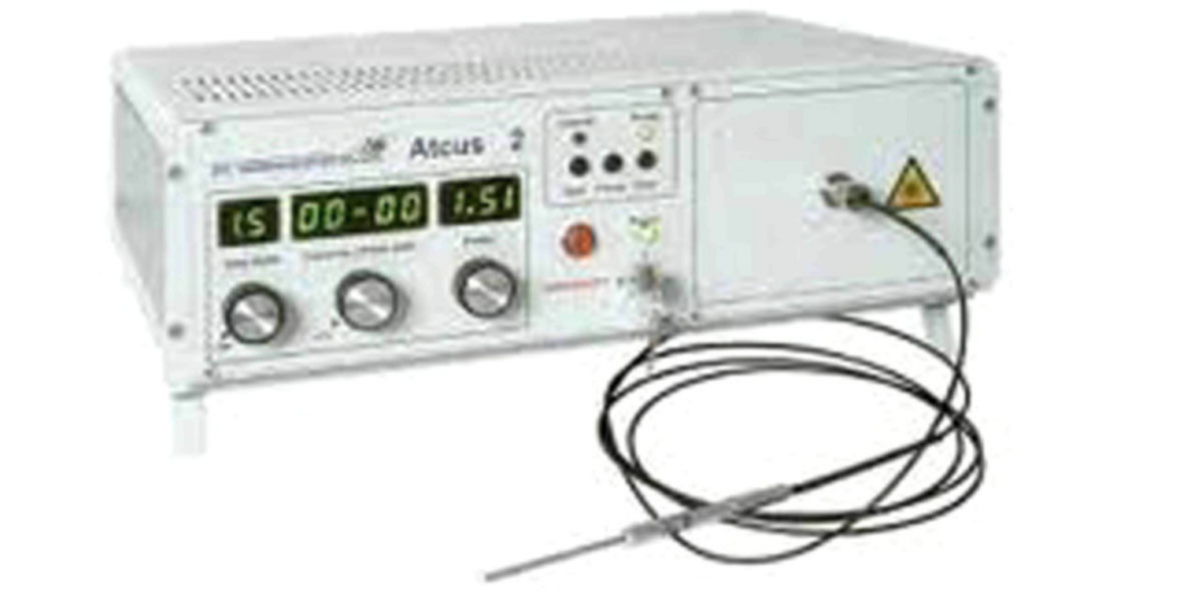
medical device ATCUS-2

1. Experimental part. Growing laboratory rats (4-8.5 months) of both sexes were subjected to the single procedure of PDI on the knee joints area with transcutaneous PS (Fotoditazin ®. See Fig. 1) administration. The medical device ATCUS-2 (ATC-"Semiconductor Devices", Russia. See Fig. 2) was used as the laser source. The weight, body length and the length of the thighs and shanks (on radiographs at standard conditions) of all the animals were measured before the experiment and until the age of 8.5 months with an interval of 2 weeks. The results were compared with similar measurements of control group animals not subjected to PDI.

2. Clinical part. 7 children of both sexes aged 12 years with progressive AIS (25°-35° by Cobb) were subjected to a single procedure of PDI on the top area of the scoliotic curve with transcutaneous PS (Fotoditazin ®) administration. Subsequent development of scoliotic curve was evaluated during 18 months by spondyloradiographs in direct rear projection and compared with similar studies in the control group patients with progressive AIS not subjected to PDI.

## Results

1. The animals of the experimental group showed a slowdown in the hips and legs for 1.5 months after PDI, followed reclaimed normal growth.

2. The slowing of the spinal deformity progression was found in children subjected to PDI.

## Conclusion

1. PDI with a transcutaneous administration of PS on the area of GP reversibly inhibits the growth of long bones in growing animals.

2. First clinical experience of PDI as a non-invasive method of progressive AIS treatment shows encouraging results.

